# Quantification of Amlodipine Maleate Content in Amorphous Solid Dispersions Produced by Fluidized Bed Granulation Using Process Analytical Technology Tools

**DOI:** 10.3390/pharmaceutics16121538

**Published:** 2024-12-01

**Authors:** Sandi Svetič, Laura Medved, Klemen Korasa, Franc Vrečer

**Affiliations:** 1 KRKA, d. d., 8501 Novo Mesto, Slovenia; sandi.svetic@krka.biz (S.S.); laura.medved@krka.biz (L.M.); klemen.korasa@krka.biz (K.K.); 2 Faculty of Pharmacy, University of Ljubljana, 1000 Ljubljana, Slovenia

**Keywords:** amorphous solid dispersion, process analytical technology, near-infrared spectroscopy, Raman spectroscopy, fluidized bed granulation, multivariate analysis

## Abstract

**Background:** Active pharmaceutical ingredient (API) content is a critical quality attribute (CQA) of amorphous solid dispersions (ASDs) prepared by spraying a solution of APIs and polymers onto the excipients in fluid bed granulator. This study presents four methods for quantifying API content during ASD preparation. **Methods:** Raman and three near-infrared (NIR) process analysers were utilized to develop methods for API quantification. Four partial least squares (PLS) models were developed using measurements from three granulation batches, with an additional batch used to evaluate model predictability. Models performance was assessed using metrics such as root mean square error of prediction (RMSEP), root mean square error of cross-validation (RMSECV), residual prediction deviation (RPD), and others. **Results:** Off-line and at-line NIR models were identified as suitable for process control applications. Additionally, at-line Raman measurements effectively predicted the endpoint of the spraying phase. **Conclusions:** To the best of authors’ knowledge, this is the first study focused on monitoring API content during fluidized bed granulation (FBG) used for ASD preparation. The findings provide novel insights into the application of Raman and NIR process analysers with PLS modelling for monitoring and controlling ASD preparation processes.

## 1. Introduction

A large percentage of marketed drugs (~40%), as well as those in development (~90%), exhibit poor aqueous solubility [1]. Drug candidates with low solubility present challenges, such as reduced bioavailability and a higher likelihood of being removed from the development process [2]. The preparation of amorphous solid dispersions (ASDs) is a formulation strategy used for improving the bioavailability of poorly soluble active pharmaceutical ingredients (APIs). This approach involves dispersing APIs in a polymer matrix. ASD formulations improve the bioavailability of the APIs by enhancing its apparent solubility and dissolution rate. These improvements result from the higher free energy, along with the increased thermodynamic and chemical activity, of amorphous APIs compared to their crystalline counterparts [3].

The preparation of an ASD using fluidized bed granulation (FBG) is categorised as a solvent-based method, where the API is first dissolved in an organic or aqueous solvent, then sprayed onto the fluidized excipients, and simultaneously dried. During this process, ASD is layered onto the excipient cores [1,4,5]. One of the most important critical quality attributes (CQAs) of the granules produced by spraying the API solution is the API content. Various factors can cause deviations from the expected API content. For instance, granulation liquid may be sprayed onto the chamber walls, or droplets may be spray-dried and carried through the filters, leading to lower API content. Conversely, the API content may be higher than expected due to selective excipient loss through the distribution plate or filters. Therefore, achieving the target API content can be challenging when the only parameter used to estimate API content is the amount of granulation liquid sprayed.

In 2002, the United States Food and Drug Administration (FDA) addressed the issue of pharmaceutical processes being monitored and controlled solely by focusing on process parameters, without real-time insight into the status of CQAs. The initiative, titled “Pharmaceutical Current Good Manufacturing Practices (CGMPs) for the 21st Century”, aimed to ensure quality is built into the product throughout the manufacturing process [6]. The key tool of the initiative was process analytical technology (PAT), defined as a system for designing, analysing, and controlling manufacturing through the timely measurements of critical performance attributes (CPAs) and CQAs of processes and in-process materials. Measurements are performed using process analysers, which capture the data in real or near real time. The measurements can be performed: in-line (the sample is measured during processing), on-line (the sample is removed from the process, measured, and possibly returned), and at-line (the sample is removed from the process and analysed nearby). Analysis conducted in a laboratory setting is referred to as off-line analysis [7].

Both near-infrared (NIR) spectroscopy and Raman spectroscopy are commonly used PAT analysers that allow fast, cost-effective, and non-destructive measurement collection. The resulting spectra provide information on both the physical and chemical properties of the analysed materials [8,9].

There is an existing body of literature on the application of NIR spectroscopy for quantification of the API content during FBG. Gavan et al. used an NIR analyser for in-line monitoring of total polyphenolic content (TPC) during the process of spraying *A. genevensis* liquid extract onto a mixture of lactose and microcrystalline cellulose (MCC) in a fluidized bed (FB) processing chamber. An orthogonal partial least squares (OPLS) model was developed to predict TPC from the NIR spectra, achieving an R^2^ of 0.982 for correlated variability and a Q^2^ of 0.951. The root mean square error of cross-validation (RMSECV) was 31.5 mg gallic acid per 100 g dry extract, while the finished dry extract contained 469.25 mg of gallic acid per 100 g dry extract. The authors noted that the developed method detected the process endpoint when the desired TPC concentration was reached [10]. Roggo et al. applied an in-line NIR probe to monitor the API content in the granulate exiting the FB processing chamber and in the tablet press feed frame. The specific API used was not disclosed. A single partial least squares (PLS) model was developed, achieving R^2^ of 0.996 and RMSECV of 1.25% [11]. Zhao et al. developed multiple models for monitoring the content of three APIs (albiflorin, paeoniflorin, and benzoylpaeoniflorin) from NIR spectra collected off-line from granulate samples. These three APIs were present in the herbal extract, which was sprayed onto maltodextrin during the FBG process. PLS, the genetic algorithm interval PLS (GA-iPLS), back-propagation artificial neural network (BP-ANN), and particle swarm optimisation support vector machine (PSO-SVM) models were developed, with the best-performing PSO-SVM models achieving R^2^ values between 0.978 and 0.985, and root mean square error of prediction (RMSEP) values of 0.0947 (albiflorin), 0.2850 (paeoniflorin), and 0.0134 (benzoylpaeoniflorin) [12]. Zhong et al. applied in-line NIR spectrometry to monitor nifedipine content uniformity during FBG, where a granulation liquid containing a binder was sprayed onto a mixture of nifedipine, MCC, and lactose. PLS and extended iterative optimisation technique (EIOT) models were developed, achieving R^2^ values of 0.982 and 0.968, Q^2^ values of 0.612 and 0.885, and RMSEP values of 3.40% and 2.77%, respectively [13].

To the best of the authors’ knowledge, no studies have focused on the quantification of API content during FBG using Raman spectroscopy. However, there are studies that have applied Raman spectroscopy for API quantification during other pharmaceutical processes. Harting and Kleinebudde applied in-line Raman spectroscopy to twin-screw wet granulation for the quantification of ibuprofen and diclofenac sodium. Separate PLS models were developed for each API. The ibuprofen model achieved a Q^2^ of 0.993, and an RMSEP of 0.59%, while the diclofenac sodium model achieved a Q^2^ of 0.997, and an RMSEP of 1.50% [14]. In a subsequent study, they optimised the in-line Raman setup by including interior lighting. They developed “dark” and “light” PLS models for diclofenac sodium prediction, with R^2^ values of 0.975 and 0.923, Q^2^ of 0.998 for both models, and RMSEP of 0.31% and 0.29%, respectively [15]. Müller et al. applied in-line Raman spectroscopy to monitor diprophylline content in the film coat during the tablet film coating process in a pan coater. In this process, a dispersion containing diprophylline was used for the tablet coating. Multiple PLS models were developed to predict relative (RMSEP: 0.6322%) weight gain, diprophylline content increase (RMSEP: 3.361%), and total diprophylline content (RMSEP: 0.4258 mg) in the coated tablets [16]. In subsequent studies, the monitoring method was validated according to ICH guideline Q2 and transferred from a mini scale pan coater to a micro-scale coater [17,18].

The aim of this study was to develop methods for the quantification of amlodipine maleate (AM) during the FBG process used for producing an ASD. This was achieved by using NIR and Raman PAT process analysers, capable of performing in-line and at-line measurements. The developed methods could be used for process end-point detection and real-time product release.

This article distinguishes itself from the previous research by focusing on the FBG process used to produce granulate containing an ASD, where an API and polymer solution is sprayed onto an inert powder. In contrast, earlier studies using NIR spectroscopy have mainly focused on monitoring API content during the spraying of API liquid extracts without polymers onto inert powders in a FB processing chamber [10,12], evaluating the API content in the finished granulate as it exits the chamber [11], or assessing the API content uniformity when APIs were incorporated directly into the initial powder mixture rather than being sprayed [13]. Additionally, earlier studies using Raman spectroscopy have monitored API content during twin-screw wet granulation [14,15], and tablet film coating with dispersions containing APIs [17,18], with none focusing on FBG. This article represents the first use of both Raman and NIR spectroscopy PAT analysers for determining API content within ASDs produced by FBG, as well as the first combined application of Raman and NIR spectroscopy for API content prediction during the FBG process.

## 2. Materials and Methods

### 2.1. Materials

Amlodipine maleate (AM) was supplied by Krka, d. d. (Novo Mesto, Slovenia). Polyvinylpyrrolidone, grade K-30 (PVP, marketed as Kollidon 30), was sourced from BASF SE (Ludwigshafen, Germany). Microcrystalline cellulose, grade 101 (MCC, marketed as Vivapur^®^ 101), was sourced from JRS PHARMA GmbH & Co. KG (Rosenberg, Germany). Ethanol was supplied by Krka, d. d. (Slovenia).

### 2.2. Methods

#### 2.2.1. Granule Preparation

The target granulate formulation consisted of 19.0 *w*/*w*% AM, 19.0 *w*/*w*% PVP, and 62.0 *w*/*w*% MCC. The granulation liquid was prepared by dissolving AM and PVP in 96 *v*/*v*% ethanol by heating to 50 °C. AM and PVP together composed 14 *w*/*w*% of the granulation liquid.

The batches were prepared with a laboratory-scale FB granulator—Multilab GPCG (Glatt GmbH, Binzen, Germany), equipped with a peristaltic pump and a 1.2 mm nozzle for spraying the granulation liquid. Process conditions were as follows: inlet air temperature of 55–65 °C, inlet airflow of 50–80 m^3^/h, spray rate of 15–35 g/min, and atomizing air pressure of 1.5 bar. The granulation liquid spray rate and inlet airflow were manually adjusted during the process to maintain a target product temperature of 32–34 °C, ensuring the optimal fluidization of the product. A total of 1000 g of MCC was utilised as the starting material for the granulations.

Four experiments (A–D) were performed according to the composition in Table 1. For batch A, 20 samples were collected, while for batches B–D, 22 samples were collected for each. Samples were collected during the spraying phase of the process. The interval between sample collections was approximately 6 min. Sample names are combinations of the batch letter (e.g., A for batch A) with the sample number, resulting in labels such as A-1.

#### 2.2.2. Determination of Amlodipine Maleate Content

The AM content in each granulate sample was determined using UV-VIS spectroscopy. A 500 mg sample of granulate was placed in a 100 mL flask, which was then filled to the mark with ethanol. The samples were mixed for 30 min using magnetic mixers, followed by 15 min in an ultrasonic bath. Afterwards, they were centrifuged for 10 min at 3500 rpm. A 5 mL aliquot of the centrifuged solution was transferred to a 50 mL flask and then filled to the mark with 0.001 M HCl. The absorbance was measured at a wavelength of 366 nm using the Agilent 8453 UV-VIS spectrometer (Agilent Technologies, Santa Clara, CA, USA). Instrument management and data logging were carried out using a UV/Visible GLP Chemstation rev. B.05.04 (Agilent Technologies, USA). The concentration of AM in each granulate sample was calculated from the absorbance data.

#### 2.2.3. X-Ray Powder Diffraction

The X-ray powder diffraction (XRPD) analysis was performed on an X’Pert PRO MPD instrument (Malvern Panalytical, Malvern, UK), using CuKα radiation (λ = 1.5406 Å), 45 kV and 40 mA. Instrument management and data logging were carried out using Data Collector 7.4 (Malvern Panalytical, UK). Data were collected in step-scan mode with a step size of 0.033° in 2θ over an angular range of 4–30 ° in 2θ, using an X-Celerator detector (Malvern Panalytical, Malvern, UK). Analysis was performed on samples C-22, AM, and MCC.

#### 2.2.4. Scanning Electron Microscopy

A scanning electron microscope (SEM) (Carl Zeiss Microscopy GmbH, Jena, Germany) was used to image crystalline AM (CAM), sample A-20 (containing 18.8% amorphous AM (AAM)), and sample A-19 spiked with CAM (containing 17.5% AAM and 1.6% CAM). Imaging was performed at an accelerating voltage of 1.00 kV, with a magnification of 1000×. The instrument management and data logging were carried out using ZEISS SmartSEM 5.07 (Carl Zeiss AG, Baden-Württemberg, Germany).

#### 2.2.5. Preliminary Raman and NIR Analysis of Materials

Raman and NIR spectroscopy were employed to analyse AM, PVP, and MCC. Raman measurements were carried out with a PhAT probe (Kaiser Optical Systems, Inc., Ann Arbor, MI, USA), connected to a Raman RXN1™ Spectrometer (Kaiser Optical Systems, Inc., USA). The instrument management and data logging were carried out using HoloGRAMS 4.2 (Kaiser Optical Systems, Inc., USA). The system employed a 785 nm excitation laser with 260 mW, producing a circular illumination area with a 6 mm diameter, using the channel “integrating sphere”. The measured spectral range was from 1763 to 700 cm^−1^. A single spectrum was calculated as an average of two acquisitions with a 10 s integration time. A Fourier transform NIR (FT-NIR) Multi-Purpose Analyser (Bruker, Billerica, MA, USA) equipped with a diffuse reflectance probe was used for acquiring NIR measurements. The instrument management and data logging were carried out using OPUS 8.5 (SP1) (Bruker, USA). The spectral measurements ranged from 8000 to 4000 cm^−1^ with a resolution of 8 cm^−1^, using an “integrating sphere” channel. Each measurement consisted of 32 scans.

#### 2.2.6. Loss on Drying

The water content of the collected samples was determined through loss on drying (LOD) measurements. For this, 2 g samples were analysed using a Mettler Toledo HX204 (Mettler Toledo, Columbus, OH, USA) apparatus, following a 105 °C, 5 min heating programme. The mass difference before and after drying was expressed as the percentage of mass loss.

### 2.3. Process Analytical Technology Analysers

#### 2.3.1. At-Line Raman Spectroscopy

At-line Raman measurements were conducted using a PhAT probe (Kaiser Optical Systems, Inc., USA) connected to a Raman RXN1™ Spectrometer (Kaiser Optical Systems, Inc., USA). The system utilises a 785 nm excitation laser with a power output of 260 mW, creating a circular illumination area with a 6 mm diameter. The spectral range measured was from 1763 to 700 cm^−1^. The “integrating sphere” channel was used for the measurements. Each spectrum was obtained by averaging two acquisitions, with an integration time of 10 s. The instrument management and data logging were carried out using HoloGRAMS 4.2 (Kaiser Optical Systems, Inc., USA).

#### 2.3.2. Off-Line NIR Spectroscopy

A FT-NIR Multi-Purpose Analyser (Bruker, USA) equipped with a diffuse reflectance probe was used for off-line NIR measurements. The spectral range applied was 6900–3800 cm^−1^, with a resolution of 8 cm^−1^, using the “integrating sphere” channel. Each measurement involved 32 scans. For every sample, two measurements were taken and averaged before multivariate analysis. The instrument management and data logging were carried out using OPUS 8.5 (SP1) (Bruker, USA).

#### 2.3.3. At-Line NIR Spectroscopy

A diffuse reflectance NIR spectrometer Antaris™ Target Analyser (Thermo Fisher Scientific, Waltham, MA, USA) was used for at-line NIR process measurements. The applied spectral range was 7390–5580 cm^−1^, with a resolution of 8 cm^−1^. Each measurement consisted of 32 scans. For each granulate sample and spiked granulate sample, four parallel measurements were taken and averaged before performing the multivariate analysis. The NIR probe and data logging were managed using RESULT 3 SP8 (Thermo Fisher Scientific Inc., USA).

#### 2.3.4. In-Line NIR Spectroscopy

In-line NIR measurements were conducted using a diode array NIR spectrometer (J&M Analytik AG, Essingen, Germany) equipped with a diffuse reflectance probe (Lighthouse probe™, GEA Pharma Systems, Wommelgem, Belgium). The probe was installed through the lowest window in the FB processing chamber, which is the closest to the sampling port level. The measurements utilised a resolution of 4 nm, covering a spectral range of 8750–4850 cm^−1^. The NIR probe and data logging were managed using SinTQ™ 3.4 (Optimal Industrial Automation, Bristol, UK). Measurements were taken every 10 s, with an integration time of 95 ms for each spectral measurement.

### 2.4. Multivariate Data Analysis

Aspen Unscrambler^®^ 12.2 (Aspen Technology, Inc., Bedford, MA, USA) was used for spectral pre-treatment and multivariate data analysis. Four PLS models were trained using data from batches A, B, and C, resulting in models ABC1–4. The letters indicate batches used for training, and the number denotes the probe used: ABC1 (at-line Raman), ABC2 (off-line NIR), ABC3 (at-line NIR), and ABC4 (in-line NIR). The output for models was the AM concentration. The wavenumber ranges and applied transformations were iteratively optimised to enhance prediction performance. Table 2 summarises the applied spectral ranges and pre-processing methods. The cross-validated ABC1 model was used in combination with at-line measurements for end-point detection in batch D. This model was selected for its strong predictive power among the cross-validated models, with only the ABC2 NIR model outperforming it; however, the ABC2 model required off-line analysis. Additionally, the external validation of all models was performed using data from batch D, which was not included in the training set.

#### Model Evaluation

Models were evaluated using multiple metrics: RMSECV, RMSEP, residual prediction deviation (RPD), bias, R^2^ (cross-validation), Q^2^ (external validation), and detection limit (DL). Concentrations of AM, expressed as percentages, were outputs of the models. RMSECV, RMSEP, bias, and LOD are also expressed as a percentage. Additionally, to assess the error relative to the total API concentration (TAC), RMSECV/TAC and RMSEP/TAC were calculated. The predictive power of the models was evaluated using the RPD metric. A commonly accepted rule is that an RPD above 3.00 indicates a good model. This can be further expanded into a scale where values below 2.3 signify a very poor model, 2.4–3.0 indicate a poor model suitable for rough screening, 3.1–4.9 suggest a fair model useful for screening purposes, 5.0–6.4 represent a good model useful for quality control, 6.5–8.0 signify a very good model suitable for process control, and an RPD above 8.1 indicates an excellent model suitable for any application [19,20]. Additionally, predicted vs. reference charts were used to identify patterns in the differences between predicted and reference values, alongside the quantitative metrics listed above [21].

To evaluate the models, the European Medicines Agency (EMA) guideline specifying an acceptable maximum acceptable deviation (MAD) of ±5% (95–105%) for API content in finished products was used [22]. The RMSEP represents a model error that can be either positive or negative. Narrower MAD limits were calculated by subtracting the RMSEP from the guideline limits. The feasibility of these narrower limits was assessed for all models, along with an evaluation of other relevant metrics.

## 3. Results

### 3.1. Evaluation of ASD Formation

ASD formation was evaluated using two methods: XRPD, the most commonly used technique for this purpose, and SEM, which can be used to detect surface crystallinity. Since ASD prepared by FBG forms a layer over MCC particles, SEM offers additional insights into the surface characteristics of the final product [23].

#### 3.1.1. X-Ray Powder Diffraction

**Figure 1:**
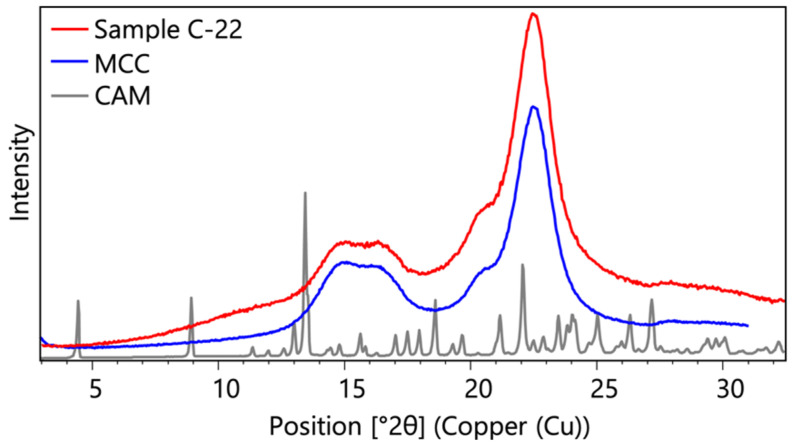
XRPD pattern of samples C-22, MCC, and CAM.

The XRPD pattern in Figure 1 displays intensity versus 2θ position for three samples: C-22, MCC, and AM. The sample C-22 shows two broad peaks with maximums around 16° and 22.5° in 2θ, closely resembling the pattern of MCC. This similarity indicates that MCC significantly contributes to the structural profile of C-22. The absence of distinct crystalline peaks in C-22, which would correspond to AM, confirms that AM is not present in a crystalline state. This analysis confirms the amorphous nature of AM in C-22, with no detectable crystalline AM phases.

#### 3.1.2. Scanning Electron Microscopy

**Figure 2:**
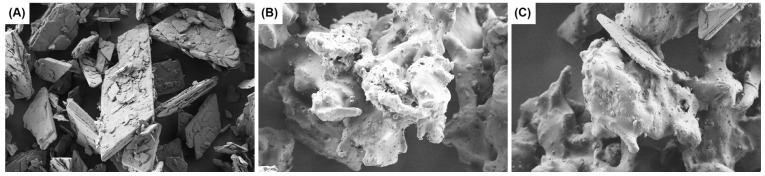
SEM images captured at 1000x magnification of (**A**) CAM, (**B**) sample A-20 (containing 18.8% AAM), and (**C**) granulate sample A-19 spiked with CAM (containing 17.5% AAM and 1.6% spiked CAM).

ASD was further evaluated using SEM imaging to detect trace crystallinity on the surface of the granules. Initially, the morphology of pure CAM was recorded to establish its appearance (Figure 2A). Subsequently, a final sample from batch A, labelled A-20, which was expected to contain only AAM without crystalline particles, was imaged (Figure 2B). As a control, the batch A sample A-19 was spiked with 1.6% CAM to demonstrate the appearance of CAM particles in granulate samples if residual CAM were present (Figure 2C). In Figure 2A, the CAM appears as large, flat particles, which are also visible in the spiked control sample in Figure 2C. In contrast, Figure 2B (sample A-20) shows no large, flat CAM particles. Multiple images of sample A-20 were obtained to confirm the absence of residual crystallinity.

### 3.2. Preliminary Raman and NIR Analysis

In the preliminary Raman and NIR analysis, CAM, AAM, PVP, and MCC were analysed to determine if the resulting spectra provided a sufficiently robust basis for predictive modelling, enabling the monitoring of AM concentration during FBG. Each formulation component (CAM, AAM, PVP, and MCC) was separately analysed with Raman and NIR spectroscopy to check if AM signals would overlap with those of the other components.

**Figure 3:**
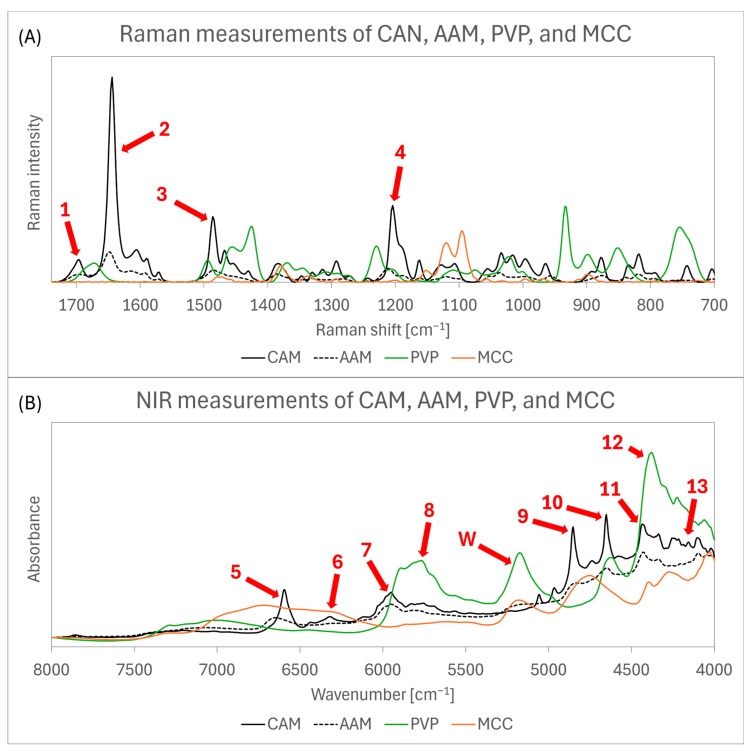
(**A**) Raman and (**B**) NIR spectra of CAM, AAM, PVP, and MCC. The red arrows indicate the most significant Raman peaks and NIR bands, as detailed in Section 3.2. Arrow 1 (1690 cm^−1^), arrow 2 (1640 cm^−1^), arrow 3 (1480 cm^−1^), arrow 4 (1200 cm^−1^), arrow 5 (6590 cm^−1^), arrow 6 (6300 cm^−1^), arrow 7 (5980 cm^−1^), arrow 8 (5700–5900 cm^−1^), arrow 9 (4860 cm^−1^), arrow 10 (4660 cm^−1^), arrow 11 (4440 cm^−1^), arrow 12 (4370 cm^−1^), arrow 13 (4270–4060 cm^−1^), and arrow W (5170 cm^−1^).

Raman spectra are displayed in Figure 3A. The first peak at 1690 cm^−1^ (arrow 1), corresponding to AM, partially overlapped with PVP peak at 1670 cm^−1^. The most prominent peak at 1640 cm^−1^ (arrow 2), corresponding to AM, did not overlap with the signals from either PVP or MCC. PVP signals partially overlapped with AM peaks at 1480 cm^−1^ (arrow 3) and 1200 cm^−1^ (arrow 4). Preliminary analysis suggests that this composition provides a strong foundation for developing a predictive model used to estimate AM concentration. However, a potential challenge arises due to the amorphous state of the API, which shows much less intense peaks compared to CAM.

NIR spectra are displayed in Figure 3B. AM bands are visible at 6590 cm^−1^ (arrow 5), 6300 cm^−1^ (arrow 6), 5980 cm^−1^ (arrow 7), 4860 cm^−1^ (arrow 9), 4660 cm^−1^ (arrow 10), 4440 cm^−1^ (arrow 11), and within the range of 4270 to 4060 cm^−1^ (arrow 13). However, some of these bands are at least partially overlapped with MCC bands, which could negatively impact the predictive power of models based on the NIR spectra. Additionally, there is partial overlap between PVP band at 5700 to 5900 cm^−1^ (arrow 8) and the AM band at 5980 cm^−1^ (arrow 7), as well as between the PVP band at 4370 cm^−1^ (arrow 12) and the AM band at 4440 cm^−1^ (arrow 11). However, since the concentrations between AM and PVP are correlated, this overlap is unlikely to have a negative effect on the models’ predictive power. Furthermore, a water band is visible at 5170 cm^−1^ (arrow W) in both PVP and MCC spectra [24]. This is expected, as MCC contains between 3.0 and 5.0% water and PVP contains up to 5.0% water [25,26]. Similarly to the Raman spectra, AAM bands show much lower intensity compared to CAM spectra, which may make the prediction of AM concentration in ASD more challenging.

### 3.3. Batch Comparability Assessment

Variability between the spectra of all four batches was analysed using Principal Component Analysis (PCA) [9,27]. Variability within the training datasets is particularly important, as it supports the development of more robust models. Ideally, training datasets should include both upper and lower limit values of process parameters, establishing the range within which the model can reliably make predictions [28]. If the granulation processes were identical across all batches, samples would vary only in AM and PVP concentrations, resulting in PCA score plots with all points aligned along a single process trajectory. However, if additional variability exists among batches—such as differences in experimental conditions affecting the spectra—multiple distinct process trajectories would appear in the PCA plots [9].

#### 3.3.1. PCA of Raman Spectra

**Figure 4:**
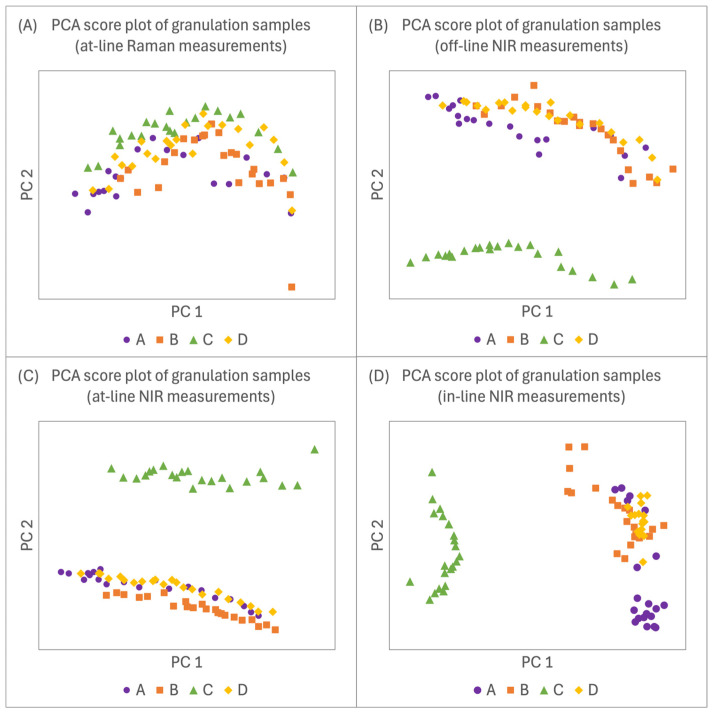
PCA score plots of NIR and Raman spectra of batches (**A**–**D**).

The PCA score plot for at-line Raman measurements in Figure 4A shows a similar trend across all four batches with a clear differentiation among them. The process trajectory for batch D is positioned roughly in the centre of the three batches used in the training dataset, indicating that the variability within the training dataset is sufficient for predicting AM concentrations in batch D samples. An outlier was identified in the lower right corner of the score plot, corresponding to B-1 sample from batch B. This may have resulted from unrepresentative sampling or measurement error. Consequently, the outlier data point was excluded from the training dataset. To minimise the occurrence of outliers in the future, samples should be homogenised prior to analysis, multiple measurements should be taken from different parts of the sample, and spectra should be carefully inspected to allow for the re-analysis of any samples with irregularities.

#### 3.3.2. PCA of NIR Spectra

**Figure 5:**
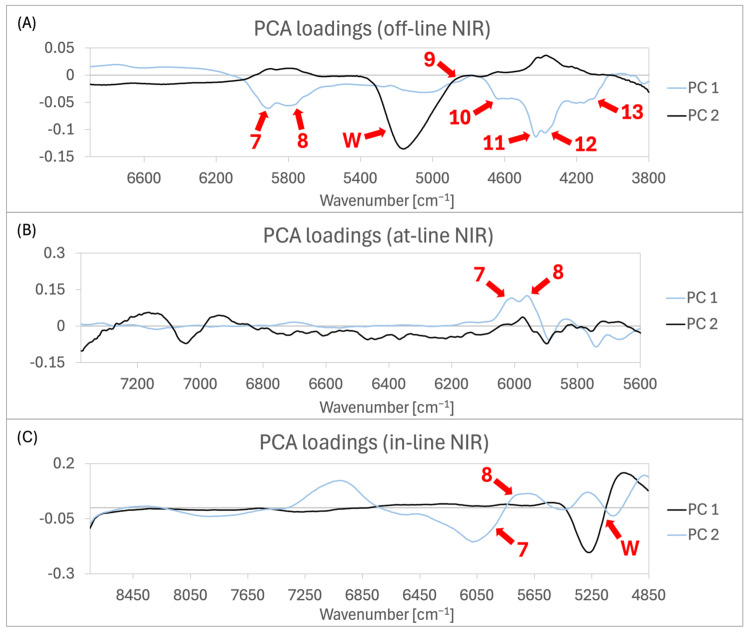
PCA loadings plots for (**A**) off-line, (**B**) at-line, and (**C**) in-line NIR data of batches A–D, with red arrows highlighting key bands for the PC where the biggest differences between batch C and the other batches are visible in the PCA score plot. Arrow 7 (5980 cm^−1^), arrow 8 (5700–5900 cm^−1^), arrow 9 (4860 cm^−1^), arrow 10 (4660 cm^−1^), arrow 11 (4440 cm^−1^), arrow 12 (4370 cm^−1^), arrow 13 (4270–4060 cm^−1^), and arrow W (5170 cm^−1^).

The score plots of all three NIR analysers are shown in Figure 4B–D, indicating that the samples from batch C significantly differed from those of batches A, B, and D. Loadings plots for the three PCA analyses were examined, focusing on the PCs (principal components) that displayed visible differentiation between the two clusters: PC1 for in-line NIR and PC2 for off-line and at-line NIR (Figure 5).

The loadings plot of off-line NIR spectra in Figure 5 shows that the highest loadings in PC2 are around the wavenumber 5200 cm^−1^, corresponding to the water bands. Analysis of the loadings plots for the at-line and in-line NIR spectra in Figure 5A,B is more complex due to the pre-processing of the original spectra using Savitzky–Golay first-order derivative transformation. In the PC2 loading plot of at-line NIR spectra in Figure 5B, the highest weighted bands between 6840 and 7380 cm^−1^ likely correspond to the water band, typically found between 6880 and 7020 cm^−1^ [24]. The PC1 loadings plot for the in-line NIR spectra shows weighed bands at 5250 and 5260 cm^−1^, the first is positive and the second is negative, suggesting the presence of a single water absorption band in the spectra prior to pre-processing.

**Figure 6:**
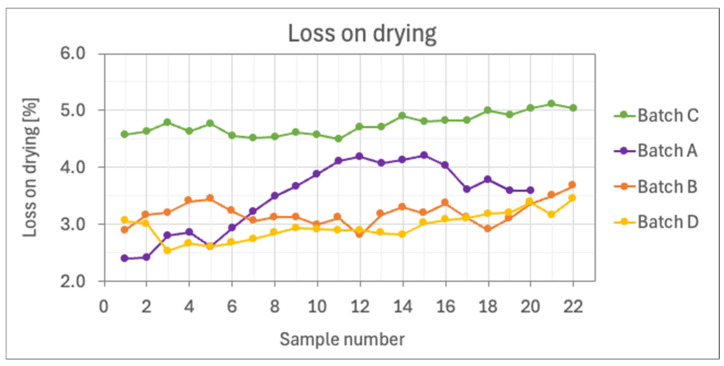
LOD measurements of the collected samples.

Additionally, process parameters were examined to clarify the differences observed with NIR spectroscopy. Batches A, B, and D had similar inlet air humidity levels, ranging from 1.6 to 2.3 g/kg, while batch C exhibited much higher inlet air humidity, ranging from 1.9 to 9.2 g/kg. Outlet air humidity levels were also consistent for batches A, B, and D, at between 2.1 and 3.7 g/kg, whereas batch C showed significantly higher values, ranging from 6.9 to 8.5 g/kg. The LOD measurements for MCC used in processing were comparable: 4.04 (batch A), 4.12 (batch B), 4.16 (batch C), and 4.08 (batch D). The LOD measurements of collected samples, shown in Figure 6, confirm that the samples from batch C generally displayed higher LOD values, confirming that these differences are due to variations in inlet air humidity levels during FB processing. It is worth mentioning that having higher moisture content in a portion of the training set samples does not diminish the effectiveness of the final model. Variability in water content among training samples could impact the model’s predictive performance if those bands carry significant weight in the final models. However, if the resulting models display low loading values for wavenumbers associated with water, it indicates that the models have effectively accounted for varying water levels without affecting the AM concentration predictions [29].

### 3.4. Modelling Amlodipine Maleate Concentration

Four PLS models were developed using measurements from batches A, B, and C as the training dataset, while batch D measurements were used for external validation. In the subsequent subsections, the loadings for each model were analysed in conjunction with the sample spectra. Additionally, the model parameters, presented in Table 3, were reviewed to assess performance and suitability.

#### 3.4.1. Analysis of Spectra and Loadings

Batch A spectra were analysed alongside model ABC1-4 loadings to identify which parts of the spectrum had the greatest influence on the predictions. This analysis was performed to confirm that the weighted regions of the spectrum aligned with peaks and bands expected to vary with changes in AM concentration [30]. The charts display model loadings in the top section (e.g., factor 1—loading) and the spectral measurements in the bottom section. The explained variances in Table 4 show that for models ABC1-3, factor 1 captures over 96% of the covariance between the spectra and AM concentrations, while for model ABC4, factor 1 captures around 84% of the covariance. Since factor 1 accounts for the majority of the covariance in all models, only its loadings were analysed.

##### Model ABC1 (At-Line Raman)

**Figure 7:**
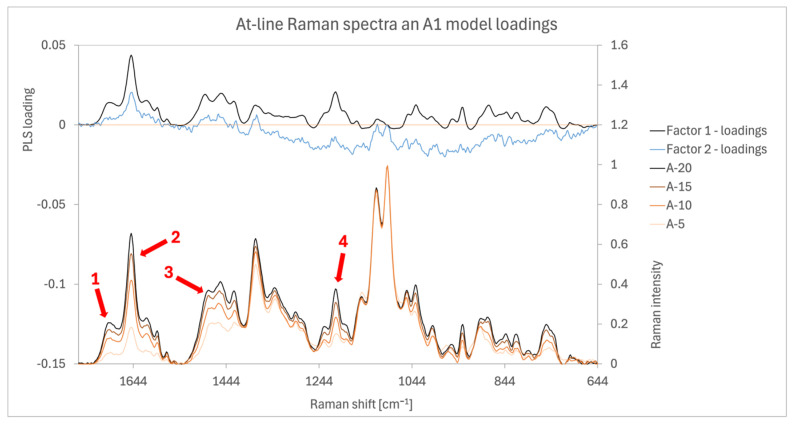
Spectra and loadings chart of the at-line Raman model A1 with red arrows highlighting the most prominent peaks that show signal differentiations between the different samples. Arrow 1 (1690 cm^−1^), arrow 2 (1640 cm^−1^), arrow 3 (1480 cm^−1^), and arrow 4 (1200 cm^−1^).

The most significant spectral variations between the samples, shown in Figure 7, occur between peaks corresponding to AM and PVP. Specifically, these variations are observed at the following Raman shifts: 1690 cm^−1^ (arrow 1, attributed to AM according to Figure 3A), 1640 cm^−1^ (arrow 2, AM), 1480 cm^−1^ (arrow 3, AM), 1450 cm^−1^ (PVP and AM), 1425 cm^−1^ (PVP and AM), between 1250 and 1180 cm^−1^ (arrow 4, PVP and AM), 1050 cm^−1^ (MCC and AM), 1030 cm^−1^ (AM and PVP), 1000 cm^−1^ (PVP and AM), 930 cm^−1^ (PVP), region between 910 and 800 cm^−1^ (AM, PVP, MCC), and 750 cm^−1^ (PVP and AM). Factor 1 loadings have high weights at all these peaks. The presence of weighted peaks for both AM and PVP aligns with the process, as the granulation liquid containing both is applied during granulation, leading to a simultaneous increase in their concentrations.

The two highest peaks in the spectra at 1100 and 1120 cm^−1^ correspond to MCC, as shown in Figure 3A. MCC concentration is inversely proportional to AM and PVP concentrations, but its peak intensity remains stable, likely due to its high concentration. The factor 1 loadings for these MCC peaks are close to zero.

##### Model ABC2 (Off-Line NIR)

**Figure 8:**
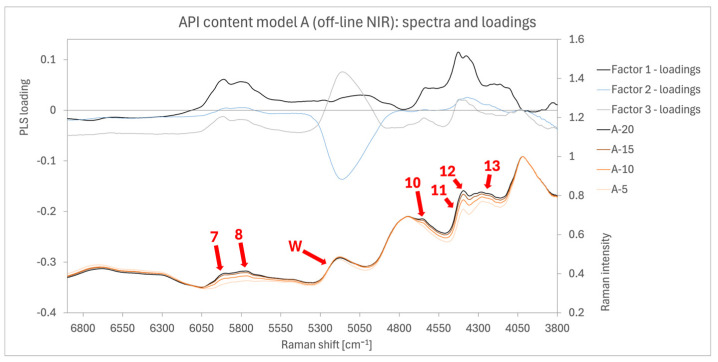
Spectra and loadings chart of the off-line NIR model A2 with red arrows highlighting the most prominent bands that show signal differentiation between the different samples. Arrow 7 (5980 cm^−1^), arrow 8 (5700–5900 cm^−1^), arrow 9 (4860 cm^−1^), arrow 10 (4660 cm^−1^), arrow 11 (4440 cm^−1^), arrow 12 (4370 cm^−1^), arrow 13 (4270–4060 cm^−1^), and arrow W (5170 cm^−1^).

NIR spectra in Figure 8 show variability primarily between AM bands and PVP bands. Off-line NIR spectra exhibited less variation among batch samples compared to the Raman measurements discussed in the previous section. This was expected, given the lower intensity observed in the preliminary analysis, especially for the AAM. The greatest spectral variation among batch samples was observed in two specific wavenumber ranges, which were analysed alongside individual component measurements in Figure 3B. The first range, from 5270 to 6050 cm^−1^, corresponds to the AM band at 5980 cm^−1^ (arrow 7) and the PVP band between 5700 and 5900 cm^−1^ (arrow 8). The second range, from 4060 to 4700 cm^−1^, includes AM bands at 4660 cm^−1^ (arrow 10) and 4440 cm^−1^ (arrow 11) and several smaller AM bands between 4270 and 4060 cm^−1^ (arrow 13), as well as the PVP band at 4370 cm^−1^ (arrow 12). These ranges also exhibited the highest model loadings. The band at 5170 cm^−1^ (arrow W), which showed no variation among the tested samples, corresponds to water/moisture and appears in spectra of both PVP and MCC, as seen in Figure 3B. Its loading was close to zero, indicating a negligible impact on the model results. Moreover, the water band carries high weights in factor 2 and factor 3 loadings. However, as factor 2 accounts for 2.11% of the variance and factor 3 explains an additional 1.03%, both factors minimally influence the model. Their effects are somewhat offset, since the water band is weighted negatively in factor 2 and positively in factor 3.

##### Model ABC3 (At-Line NIR)

**Figure 9:**
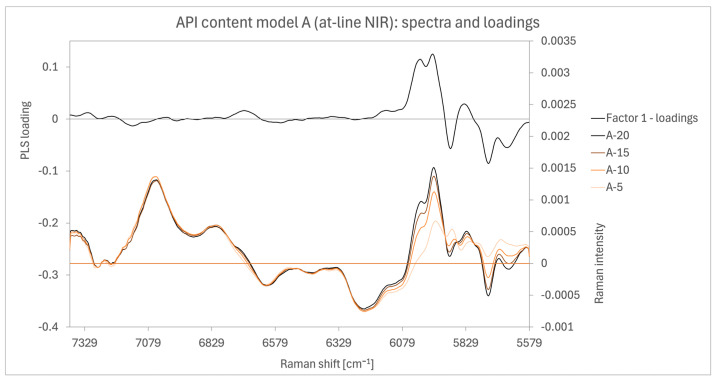
Spectra and loadings chart of the at-line NIR model A3.

The at-line NIR measurements in Figure 9 exhibited variability between samples, primarily in the spectral range corresponding to AM and PVP bands identified in the off-line NIR spectra in the previous section. The at-line NIR spectra covered a narrower range of wavenumbers, excluding the significant 4060 to 4700 cm^−1^ range but including the 5579 to 6270 cm^−1^ range. Negative absorbances were observed due to the use of Savitzky–Golay first-order derivative transformation during spectral pre-processing. In the loading chart, the weighted area between 5579 and 6270 cm^−1^ aligns with the relevant spectral range identified in the previous section.

##### Model ABC4 (In-Line NIR)

**Figure 10:**
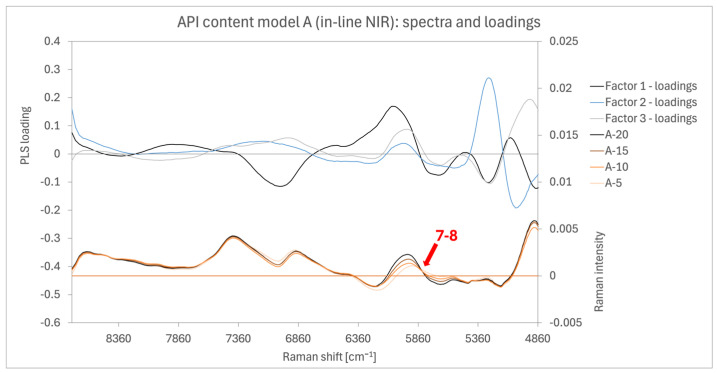
Spectra and loadings chart of the in-line NIR model A4 with red arrows highlighting the most prominent bands that show signal differentiation between the different samples. Arrow 7 (5980 cm^−1^) and arrow 8 (5700–5900 cm^−1^).

The spectra and loadings of the in-line NIR model are displayed in Figure 10. Variability between samples is observed primarily in the wavenumber ranges corresponding to AM and PVP bands. The in-line analyser covered the broadest wavenumber range but excluded the relevant 4060 to 4700 cm^−1^ region, where one of the strongest AM signals appeared in the off-line NIR spectra. Negative absorbances are a consequence of Savitzky–Golay first-order derivative transformation during spectral pre-processing. The highest spectral variability and factor 1 loading weights are observed between 5200 and 6400 cm^−1^, centred at 5830 cm^−1^ (arrow 7–8). This corresponds to the bands detected in Figure 3, specifically the AM band at 5980 cm^−1^ (arrow 7 in Figure 3) and the PVP band range of 5700 to 5900 cm^−1^ (arrow 8 in Figure 3). This variability aligns with the findings from the previous two sections on NIR measurements.

#### 3.4.2. Model Evaluation

The model metrics, shown in Table 3, and predicted versus reference charts were analysed for each model with a particular focus on their ability to predict AM concentrations within the acceptable MAD range of ±5% (95–105%). Additionally, the Raman model (ABC1) was assessed for its capability to detect the endpoint at-line during the batch D FBG process.

##### Model ABC1 (At-Line Raman)

The analysis of model parameters reveals that the Q^2^ is lower compared to R^2^, suggesting that the model explains less variability in the batch D dataset compared to the training dataset. While this is somewhat expected, it may also indicate that the training set lacks the diversity needed to accurately capture patterns in the batch D dataset. Prediction results for batch D show a significant performance drop compared to CV results, with RMSE/TAC increasing from 2.6% to 6.8%, representing the second largest prediction error within the entire set of models. Additionally, there is a noticeable increase in bias between CV and validation batch predictions, suggesting that bias is the primary factor contributing to the high RMSEP, rather than the large variability in the predictions of the granulation D dataset.

Validation parameters indicate that model ABC1 may not be sufficient to meet regulatory compliance limits, as its RMSEP/TAC of 6.8% exceeds the MAD of ±5%. With an RPD of 3.7, the model is classified as fair and useful for screening purposes. However, based on cross-validation metrics, ABC1 is the best-performing among the PAT probes, with only the off-line NIR model ABC2 outperforming it. The RMSECV/TAC value of 2.6% indicates that the model ABC1 could be used to meet the regulatory compliance limit, which is why it was employed for process endpoint detection during batch D granulation.

Subpar performance on the validation dataset could be attributed to an insufficiently representative training dataset, which may have limited its predictive accuracy on unknown measurements. To enhance the model’s predictive power, expanding the training dataset with more granulations is essential. Additionally, the calibrated concentration range may be too broad, suggesting the potential need for multiple PLS models tailored to narrower concentration ranges.

##### Model ABC2 (Off-Line NIR)

**Figure 11:**
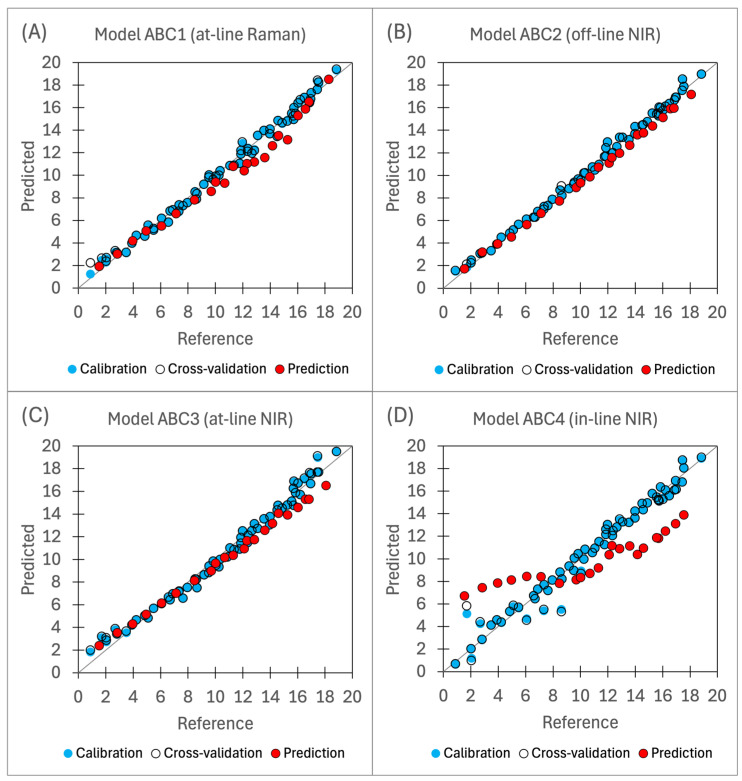
Predicted vs. reference charts for four final models: (**A**) ABC1 – at-line Raman, (**B**) ABC2 – off-line NIR, (**C**) ABC3 – at-line NIR, and (**D**) ABC4 – in-line NIR.

The model demonstrates the strongest predictive ability among the entire set, with the lowest observed prediction error RMSEP/TAC of 3.7%. R^2^ and Q^2^ values are relatively close, indicating that the model’s predictive performance on training data aligns with its performance on unseen data. The absolute value of bias is 0.60%, which is close to RMSEP at 0.7%, showing that bias is the major contributor to the RMSEP. The prediction vs. reference chart (Figure 11B) indicates good agreement between predicted and reference values.

The model ABC2 can be used to ensure compliance with regulatory API content limits, with a MAD tolerance of ±5% (95–105%). Given the model’s error (RMSEP/TAC of 3.7%), a narrow prediction limit of ±1.3% (98.7–101.3%) is necessary to meet the required endpoint concentration. This corresponds to an RPD value of 6.9, categorising the model as very good and suitable for process control. The narrow target concentration range presents a key challenge. To address this, two strategies could be used: increasing measurement frequency to capture real-time changes and reducing the spray rate as the process nears completion.

##### Model ABC3 (At-Line NIR)

The ABC3 model’s predictive ability ranks second-best, with an RMSEP/TAC of 4.8%. R^2^ and Q^2^ values are high and have similar values, indicating that the model is not overfitting the training data. The prediction vs. reference chart (Figure 11C) exhibits a slope lower than 1, indicating the presence of systemic bias in the predictions. However, the model’s bias metric has a low absolute value, as it tends to overpredict low concentrations and underpredict high concentrations. This contributes to higher RMSEP, and lower bias compared to the ABC2 model.

With an RPD of 5.2, the model is classified as good and suitable for quality control purposes. It can be used to ensure compliance with regulatory limits for the MAD of API content within ±5% (95–105%). Given the model’s RMSEP, a narrow prediction limit of ±0.2% (99.8–100.2%) should be used. As observed with the model ABC2, this narrow acceptable concentration range may pose detection challenges, where consecutive measurements may fall on either side of the limit. This challenge can be addressed using the same methods described in section: Model ABC2 (off-line NIR).

##### Model ABC4 (In-Line NIR)

The ABC4 model demonstrates the weakest predictive performance among the set, with an RMSEP/TAC of 14.9%. Its R^2^ is low and slightly below that of models ABC1-3, while the Q^2^ value is significantly lower than R^2^. This indicates overfitting to the training data and poor generalisation to new, unseen data. The predicted vs. reference chart (Figure 11D) mirrors the trend seen with the model ABC3, but with more pronounced effects. The models show over- and underprediction across different concentration ranges, resulting in a high RMSEP value despite low bias. Since the model’s error exceeds 5%, it is unsuitable for ensuring compliance with regulatory limits for the MAD of API content. With an RPD of 1.7, the model is classified as very poor and unsuitable for use. Is it important to note that while the in-line NIR analyser is the more useful PAT tool of the set due to its ability to collect real-time measurements, its predictive power is the poorest among the models.

Measurements may have also impacted prediction accuracy, as in-line measurements were not performed directly on the reference samples. Instead, reference samples were collected separately during in-line measurements, potentially leading to variations in AM concentration between the reference samples and the material analysed in-line during granulation. Additional experiments are needed to evaluate this effect, such as developing two models: one trained on spectra collected off-line from reference samples using the same probe as in-line measurements, and another trained on spectra collected in-line while reference samples were being collected, then comparing their predictive abilities. Differences in predictions between the two models would indicate the extent of this effect.

### 3.5. Process Endpoint Detection Using Raman Spectroscopy

At-line Raman probe measurements were selected as the method for endpoint detection during batch D FBG. This probe was chosen because its predictive model performed best among the PAT probes on cross-validation results, with only the ABC2 NIR model outperforming it, though that method required off-line analysis. Using an RMSECV/TAC value of 2.6%, an acceptable API content range of ±2.4% (97.6–102.4%) around the target concentration of 19% was defined, resulting in the acceptable AM concentration range of 18.5% to 19.5%. The spraying phase, during which the AM and PVP solution was applied, concluded once the first prediction reached this target range, which occurred at 18.5% during batch D. The final analysis revealed an AM content of 18.3% (96.3% of the target AM content) in the last sample, which is within the regulatory limit of ±5% (95–105 %) of the target concentration.

**Figure 12:**
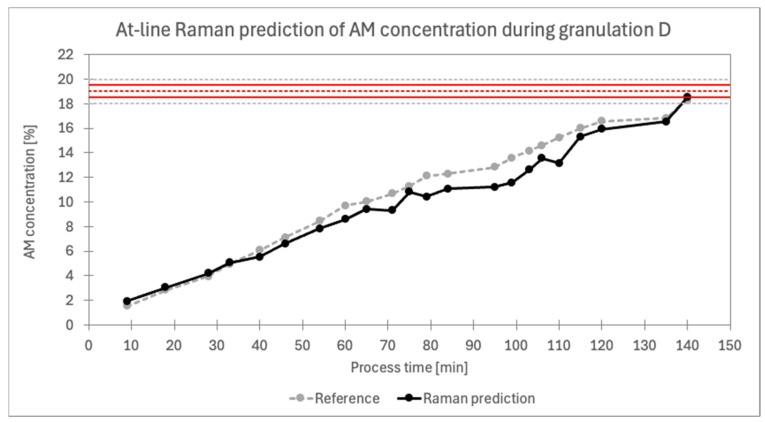
At-line Raman prediction of AM concentration during granulation D. The dashed red line represents the target AM concentration of 19%, while the two solid red lines mark the calculated endpoint limits, ranging from 97.6% to 102.4% of the target concentration, based on an RMSECV/TAC value of 2.6%. The dashed grey lines show the acceptable upper and lower limits of 95% and 105% of the target concentration.

The results in Figure 12 show Raman predictions taken during processing alongside reference values obtained off-line after the granulation. A noticeable bias appears within the 8% to 16% concentration range, as also depicted in the prediction vs. reference chart in Figure 11A. This bias suggests potential limitations in model robustness, which is consistent with the model parameter analysis in section: Model ABC1 (at-line Raman), where batch D predictions returned RMSEP/TAC of 6.8%. While a narrower RMSECV/TAC of 2.6% was used for endpoint detection, enabling compliance with the regulatory ±5% limit, these results indicate that further model optimisation would be beneficial. To improve robustness, additional granulations with deliberate variations should be conducted, and validation across multiple batches should also be performed. Additionally, models tailored to narrower concentration ranges should be developed.

## 4. Discussion

Four methods for the in-line, at-line, and off-line quantification of AM during the FBG for producing ASDs were successfully developed using Raman and NIR PAT process analysers. A key challenge in developing predictive models for ASD, compared to formulations with CAM, was the significantly weaker Raman and NIR signals associated with AAM. These differences highlight the need to develop models tailored to the specific CAM-to-AAM ratio for accurate predictions. In this study, all models were developed using a formulation containing only AAM.

Models were considered suitable for detecting AM endpoint concentrations if their RMSE/TAC values were below 5%, in line with the regulatory limit for MAD of API content. Based on RMSEP/TAC values, both the off-line and at-line NIR models met this threshold. Although the at-line Raman model, with an RMSEP/TAC of 6.8%, was deemed less suitable for process control, it was successfully used for endpoint prediction, producing results within the regulatory limit and demonstrating good predictive performance near the process endpoint. The largest deviations between predicted and reference values occurred in the middle concentration range, suggesting the need for multiple PLS models tailored to narrower concentration ranges. Differences between validation and cross-validation results indicated that the training datasets lacked sufficient variability to generalise well to new batch data. To improve model robustness, it would be necessary to expand the training dataset with additional batches.

All developed models could face challenges with narrow target endpoint concentration ranges, as this increases the risk of overshooting the desired concentration. This issue could be addressed by increasing the frequency of spectral acquisition and reducing the spray rate as the process approaches completion. Additionally, the simplified formulation in this study, containing only three components, likely aided in the detection of API peaks and bands. When developing more complex formulations, it may be necessary to be mindful of the effect components have on resulting spectra. When developing a PAT strategy for a product, it is crucial to select a combination of a PAT approaches and formulations, if possible, that allows for the detection of relevant signals.

## 5. Conclusions

During this study, methods were developed for quantifying AM during the FBG process used to produce ASDs by employing Raman and NIR PAT analysers capable of in-line and at-line measurements. These methods demonstrated the ability to detect process endpoints, addressing the challenge of weaker Raman and NIR signals associated with amorphous AM compared to its crystalline counterpart. This work represents a significant advancement over previous studies, which primarily focused on monitoring API content in simpler formulations, such as spraying API extracts without polymers or assessing API concentration in the finished granulate exiting the processing chamber. It is also the first study to combine Raman and NIR spectroscopy for API quantification during FBG, specifically for ASD production. The developed models, particularly the off-line and at-line NIR models, met regulatory thresholds for API quantification accuracy and could be adapted for industrial settings to ensure robust process control and quality assurance. By demonstrating the feasibility of real-time API monitoring in ASD formulations, this study lays a strong foundation for expanding PAT applications in pharmaceutical manufacturing.

## Figures and Tables

**Table 1:** Composition of granulate and granulation liquid.

Component	Granulation Liquid [*w*/*w*%]	Granulate [*w*/*w*%]
MCC	-	62.0
AM	7.2	19.0
PVP	7.2	19.0

**Table 2:** Used spectral ranges and pre-processing methods.

Analytical Technique	Analyser Type	Spectral Range [cm^−1^]	Pre-Processing Method
Raman	at-line	1763–700	Normalise max
NIR	off-line	6900–3800	
	at-line	7390–5580	Savitzky–Golay first-order derivative and second-order polynomial
	in-line	8750–4850	Standard normal variate (SNV) followed by Savitzky–Golay first-order derivative and second-order polynomial

**Table 3:** RMSECV, RMSEP, RMSECV/TAC, RMSEP/TAC, Bias (cross-validation—CV), Bias (P), R^2^, Q^2^, DL, and RPD of AM concentration prediction models.

Model	Analyser	Factors	RMSECV [%]	RMSEP [%]	RMSECV /TAC [%]	RMSEP /TAC [%]	BIAS (CV) [%]	BIAS (P) [%]	R^2^	Q^2^	DL [%]	RPD
ABC1	At-line Raman	3	0.4851	1.2911	2.6	6.8	0.0253	−1.0237	0.9901	0.9244	0.9	3.7
ABC2	Off-line NIR	3	0.3406	0.6944	1.8	3.7	0.0129	−0.5962	0.9951	0.9781	0.4	6.9
ABC3	At-line NIR	1	0.6098	0.9164	3.2	4.8	0.0134	−0.5944	0.9839	0.9619	1.5	5.2
ABC4	In-line NIR	3	0.9110	2.8325	4.8	14.9	0.0107	−0.1082	0.9638	0.4979	3.2	1.7

**Table 4:** Explained variances.

Analyser	Model	No. of Factors	Explained Variance Per Factor [%]			Cumulative Explained Variance [%]		
			Factor 1	Factor 2	Factor 3	Factor 1	Factor 2	Factor 3
At-line Raman	ABC1	3	96.51	1.32	1.18	96.51	97.82	99.01
Off-line NIR	ABC2	3	96.37	2.11	1.03	96.37	98.48	99.51
At-line NIR	ABC3	1	98.29	-	-	98.29	-	-
In-line NIR	ABC4	3	84.20	6.27	5.91	84.20	90.47	96.38

## Data Availability

The data presented in this study are available on request from the corresponding author.

## Abbreviations

Amorphous amlodipine maleate (AAM); amlodipine maleate (AM); active pharmaceutical ingredient (API); amorphous solid dispersion (ASD); backpropagation artificial neural network (BP-ANN); crystalline amlodipine maleate (CAM); critical performance attribute (CPA); critical quality attribute (CQA); cross-validation (CV); detection limit (DL); extended iterative optimisation technique (EIOT); European Medicines Agency (EMA); fluidized bed (FB); fluidized bed granulation (FBG); Food and Drug Administration (FDA); Fourier transform NIR (FT-NIR); genetic algorithm interval PLS (GA-iPLS); loss on drying (LOD); maximum acceptable deviation (MAD); microcrystalline cellulose (MCC); near-infrared (NIR); orthogonal partial least squares (OPLS); process analytical technology (PAT); principal component (PC); principal component analysis (PCA); partial least squares (PLS); particle swarm optimisation support vector machine (PSO-SVM); povidone K-30 (PVP); root mean square error of cross-validation (RMSECV); root mean square error of prediction (RMSEP); residual prediction deviation (RPD); scanning electron microscope (SEM); standard normal variate (SNV); total API concentration (TAC); total polyphenolic content (TPC).
